# Supplementary material for: Content-rich biological network constructed by mining PubMed abstracts
Source: BMC Bioinformatics. 2004 Oct 8;5:147. doi: 10.1186/1471-2105-5-147 (PMC528731; doi:10.1186/1471-2105-5-147)
Supplement: Additional File 5 — The original Chilibot query results of the term "long-term potentiation (LTP)" and 22 other terms, limiting the latest references analyzed to the years 1990, 1995, 2000, and 2004. [file 1471-2105-5-147-S5.bz2 › chilibotAdditionalFile5/ltp2000/html/ERK.html]

 


**ERK** (Input: ERK ) 

---


**Google Searches:** Entire Web  | EDU domain only  | PDF files only

.

**External Links:** OMIM |
LocusLink
| Swissprot
| GeneCards

  
**Maps of ERK**

Simple
Complete
graph in
radiant
tree
square
layout.

**New Hypothesis !**

**Synonyms** 

- erk   [PubMed]

**Synopsis**

- Consistent with these results, we find that **ERK** is activated by beta adrenergic receptors in CA1 pyramidal cell somas and dendrites.  Neuron, 1999 
  [23]

- Taken together, our results suggest that the peptide activate **ERK** via a G protein PI 3K Ras Raf 1 mediated signaling pathway in U937 cells.  Life Sci, 1999 
  [23]

- The results suggest that in hepatocytes a PTX sensitive mechanism, presumably involving G i proteins, exerts a stimulatory effect on **ERK** at a level distal to receptor coupling, acting either as an integral part of the signaling pathway s or by a permissive, synergistic regulation.  J Cell Physiol, 2000 
  [20]

- Thus, we further show that, in both PC12 cells and hippocampal neurons, depolarization induced calcium influx stimulates **ERK** activity in a PKA dependent manner.  J Biol Chem, 2000 
  [19]

- Furthermore, the full expression of the late phase of long term potentiation  [LTP]  L LTP and L LTP associated CRE mediated transcription requires **ERK** activation, suggesting that the activation of CREB by **ERK** plays a critical role in the formation of long lasting neuronal plasticity.  Neuron, 1998 
  [18]

- Thus, MAPK **ERK** activation is required for LTP dependent transcriptional regulation and we suggest this is regulated by two parallel signaling pathways, the MAPK **ERK** Elk 1 pathway targeting SRE and the MAPK **ERK** CREB pathway targeting CRE.  J Neurosci, 2000 
  [16]

- In addition, we found that inhibition of **ERK** activation blocked extracellular ATP stimulated DNA synthesis, thereby indicating that the **ERK** pathway mediates mitogenic signaling by P2Y receptors.  J Neurosci, 1999 
  [16]

- Given the possible role of altered gene expression in the late phase of LTP and in long term memory, we evaluated the capacity of the MAPK **ERK** extracellular signal regulated kinase to regulate phosphorylation of the transcription factor cAMP response element binding protein CREB in hippocampal area CA1.  Acta Neurobiol Exp (Wars), 2000 
  [16]

- Collectively, these results demonstrate that **ERK** MAPK activation is necessary for both memory consolidation of Pavlovian fear conditioning and synaptic plasticity in the amygdala.  J Neurosci, 2000 
  [14]

- In conclusion, our results allow to correlate the differences in the time course of **ERK** activity with the macrophagic response toward proliferation or activation.  J Biol Chem, 2000 
  [14]

- These results indicate that G i o protein, tyrosine kinase, P13K, PKC, and PLC calcium,   FEBS Lett, 1999 
  [14]

- no significant increase of DAG was found in response to EGF, neither at 3 nor at 24 h, using concentrations that markedly stimulated the **ERK** subgroup of the mitogen activated protein kinases MAPK and DNA synthesis.  J Cell Physiol, 1999 
  [12]

- The results indicate the tyrosine kinases and MAPK **ERK** are involved in hippocampal synaptic plasticity and may represent preferential intracellular targets for the actions of IL 6 in the adult nervous system.  J Neurochem, 2000 
  [11]

- Furthermore, the unglycosylated active TrkA receptors are unable to activate kinases in the Ras MAP kinase pathway, MEK and **Erk**.  J Neurobiol, 1999 
  [10]

- Similarly, the regulation of **ERK** and JNK responses involves both PLC gamma 2 and GTPases pathways.  Annu Rev Immunol, 1999 
  [10]
